# Craniofacial Involvement of Mucormycosis: A Single-Center Experience of 57 Cases From Shiraz, Iran

**DOI:** 10.1155/cjid/4780569

**Published:** 2025-07-30

**Authors:** Darioush Naddaffard, Omid Yousefi, Amirmohammad Farrokhi, Maryam Adel, Mahtab Jalali, Mina Yousefi, Reza Jalli, Reza Taheri, Mohammad Sadegh Masoudi

**Affiliations:** ^1^Department of Neurosurgery, Shiraz University of Medical Sciences, Shiraz, Iran; ^2^Department of Radiology, Shiraz University of Medical Sciences, Shiraz, Iran; ^3^School of Medicine, Fasa University of Medical Science, Fasa, Iran

**Keywords:** amphotericin, antifungal agents, cerebrovascular disorders, COVID-19, craniofacial abnormalities, endoscopic sinus surgery, invasive fungal infections, mucormycosis

## Abstract

**Background:** The incidence of mucormycosis surged significantly during the COVID-19 pandemic, particularly among patients with diabetes mellitus (DM), a history of corticosteroid use, or immunosuppression. In Iran, the heightened prevalence of this opportunistic fungal infection underscored the critical importance of timely and comprehensive treatment, encompassing both medical and surgical approaches.

**Methods and Material:** This article aims to analyze the characteristics, clinical presentations, and treatment regimens of mucormycosis patients in Iran. Data from 57 patients referred to Shiraz University of Medical Sciences, all with confirmed pathological cultures, were evaluated.

**Results:** Among these patients, 74% had pre-existing DM, and the most common symptom was periorbital edema (46%), followed by decreased visual acuity (28%). Amphotericin B was administered to 93% of the patients, while 75% received posaconazole. Surgical intervention was performed in 86% of the cases, primarily through functional endoscopic sinus surgery (FESS) (61%). Despite these efforts, the overall mortality rate was 26%.

**Conclusion:** Mucormycosis emerged as a serious complication of COVID-19 in Iran. Therefore, clinicians should include it in the differential diagnosis, particularly for patients with comorbidities or a history of antibiotic or corticosteroid use, and promptly initiate antifungal treatment and surgical intervention during potential future outbreaks.

## 1. Introduction

Mucormycosis, also known as “black fungus,” is an aggressive and often fatal fungal infection caused by Mucorales species, which present life-threatening conditions, especially in patients with underlying risk factors such as diabetes mellitus (DM), hypertension, kidney failure, organ transplant, cancer, and immunosuppression [1–3]. While mucormycosis can manifest in various forms, rhino-orbito-cerebral mucormycosis (ROCM) is the most common and severe presentation [4]. In ROCM, the infection typically begins in the oral cavity or nose and spreads to the central nervous system (CNS) through the eyes. Clinical features often include visual disturbances such as blurred vision, diplopia, or decreased visual acuity, along with symptoms like headache, facial pain, numbness, and purulent or bloody nasal discharge. Due to its angioinvasive nature in the brain, patients may experience altered mental status, coma, or cerebrovascular accidents (CVAs) [5].

During the COVID-19 pandemic, COVID-19-associated mucormycosis (CAM) emerged as a significant global health concern, particularly in regions with high rates of diabetes and widespread corticosteroid use, such as India and Iran [6–8]. COVID-19 creates a favorable environment for Mucorales spores to germinate in infected patients. This happens through multiple mechanisms, including hypoxia, hyperglycemia (exacerbated by pre-existing DM or steroid-induced), metabolic acidosis, and elevated iron levels. Another mechanism is decreased phagocytic activity of white blood cells (WBCs), either due to COVID-19 itself or as a result of steroid use, a common treatment for severe cases.

Antifungal therapy, in addition to surgical intervention following a strict treatment protocol, is direly required to face ROCM due to its high fatality rate [9]. Administration of antifungal agents such as liposomal amphotericin B, which serves as the first-line treatment, posaconazole, caspofungin, and fluconazole is crucial. In addition, surgical interventions, ranging from turbinectomy to aggressive removal of orbital contents and neurosurgical debridement, may become necessary in order to remove infected tissue and reduce fungal load [10].

Several studies, including case reports and case series, have highlighted the growing prevalence of CAM in Iran. Notable examples include a study by Fakhim et al. [11], which examined 33 patients in Tehran, and a systematic review by Nazari et al. [12].

This study presents the largest case series of CAM patients referred to Shiraz, a referral center in southern Iran, reporting the risk factors, mortality rates, and treatment regimens. These findings could provide valuable insights for improving patient outcomes and developing effective management strategies in other high-risk regions.

## 2. Materials and Methods

This study was conducted in a retrospective manner. Patients presented to the teaching hospitals under the supervision of Shiraz University of Medical Sciences, Iran, were included in this study. The initial inclusion criteria were signs and symptoms indicative of mucormycosis infection. These included periorbital edema and pain, loss of vision, colored discharge from the nasal cavity, and evidence of necrosis upon oral and nasal examination. Patients with the aforementioned symptoms, suspected of mucormycosis infection, then underwent biopsy from debridement of invaded structures and subsequent culture was sent to evaluate mucormycosis infection.

All patients suspected of infection underwent empirical antifungal therapy with intravenous amphotericin, and the decision for continuation of treatment was made based on the result of fungal culture. All included cases had a positive mucormycosis infection. Alternatively, imaging studies of patients visiting the emergency department with facial lesions were evaluated and if there was evidence of mucormycosis infection, the patient was then referred for evaluation via culture. Patients with a definitive diagnosis of mucormycosis infection then undergo daily ophthalmologist visits to evaluate disease progression, such as frozen eye and eye movement. A daily ENT visit was also performed to evaluate necrotic tissue for debridement. Intermittent neurosurgery visit was also performed to evaluate patient condition. After evaluation by multiple specialists, patients requiring surgical interventions by each service were then scheduled for surgery to control disease progression and to treat the patient further. Debridement of paranasal sinuses was performed by functional endoscopic sinus surgery (FESS) either by ENT or neurosurgery service.

The authors confirm that the ethical policies of the journal, as outlined on the journal's author guidelines page, have been strictly adhered to. The study received approval from the appropriate ethical review committee in Iran. In addition, patients or their visitors gave written informed consent for participation and dissemination at the time of admission. The plot indicated in this study has been created using GraphPad Prism Version 9 (GraphPad Software, California, USA).

## 3. Results

Overall, 57 cases (67% male) were included in this study, with a mean age of 56.03 (range: 14–80).

On admission, 48 patients presented with ophthalmic signs, accounting for 84% of the cases. The most prevalent ophthalmic sign was periorbital edema, which was the primary complaint in 26 patients (48% of the total). Following this, decreased visual acuity was reported by 28% of the patients (*n* = 16). In contrast, complete blindness was reported in 3 patients (5%). The third common ophthalmic complaint was pain in the orbital area, observed in 10 patients (18%). In addition, ptosis and proptosis were identified in 7 (12%) and 4 (7%) patients, respectively.

**Figure 1:**
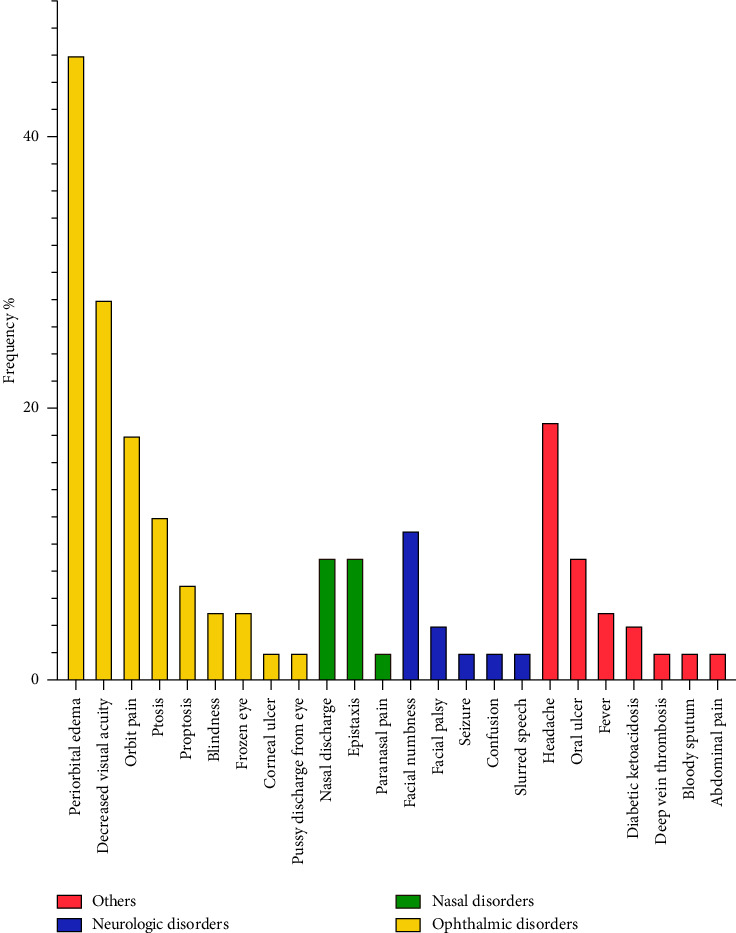
Frequency of complains on arrival per patients.

Issues related to the nasal area were present in 19% of the patients (*n* = 11) and included nasal discharge in 5 patients (9%), epistaxis in 5 patients (9%), and paranasal pain in one patient (2%). Furthermore, neurologic signs were observed in 18% of the patients, with facial numbness affecting 6 patients (11%), facial palsy in 2 patients (4%), and individual occurrences of seizure, confusion, and slurred speech in 1 patient each (2%). Notably, headache was another frequent complaint that was seen in 11 patients (19%), but it could not be categorized solely as orbital, neurologic, or nasal due to its multifactorial nature.  Table 1 and Figure 1 represent the frequency of the initial symptoms of the patients.

In our analysis of symptomatology, which is shown in Table 2, 42 patients exhibited symptoms consistent with rhino-orbital clinical syndrome (74%), 14 patients fell into the category of rhino-orbital-cerebral clinical syndrome (25%), and only one patient presented solely with symptoms indicative of sinusitis clinical syndrome (2%).

Diabetes emerged as the primary pre-existing comorbidity, existing in 74% of the patients (*n* = 42). Hypertension was also noted in 27 patients (47%). The third most frequent risk factor was ischemic heart disease, observed in 10 patients (18%).

Among the patients, six (11%) had recently consumed corticosteroids prior to admission. While one of them had rheumatoid arthritis, the other seven patients had used corticosteroids as an adjuvant therapy for COVID-19. In addition, 5 patients (9%) had a history of cancer and another 5 patients (9%) had kidney problems, including single kidney, kidney transplant, and kidney injury—all significant pre-existing comorbidities associated with mucormycosis. It is notable to say that nine patients (14%) had no pre-existing comorbidities at all. The full list of pre-existing comorbidities and their frequencies is provided in Table 3.

As it is indicated in Table 4, 93% of the patients received intravenous amphotericin, the first-line antifungal medication for mucormycosiss. Four patients did not receive amphotericin: three died before starting the medication and one declined to continue treatment and was discharged from the hospital. Followed by amphotericin, 74% of the patients received intravenous posaconazole, which is recommended in most protocols as additional therapy for mucormycosis. In addition, 21% of the patients received intravenous caspofungin. Other medications are listed in Table 4.

As indicated in Table 5, 75% of the patients underwent sinus and nasal surgical interventions, including FESS (61%), maxillectomy (16%), ethmoidectomy (11%), middle turbinectomy (7%), and nasal polypectomy (5%). Therefore, FESS was the most frequent surgical intervention. In addition, 7 patients (12%) required orbital surgeries. Of these, one patient underwent both orbitotomy and vitrectomy, while the remaining 6 patients had orbitotomy alone. Retrobulbar amphotericin injection was indicated in 28% of the patients. Finally, 8 patients (14%) did not undergo any surgical intervention.

Table 6 presents the incidence of major complications observed during hospitalization, with CNS extensions being predominant. Specifically, there were 4 cases of CVA, 3 cases of cerebral venous thrombosis (CVT), 2 cases of subarachnoid hemorrhage (SAH), and 2 cases of newly formed aneurysm. In addition, singular cases of intraventricular hemorrhage (IVH), carotid cavernous fistula, cavernous sinus thrombosis (orbital apex stenosis), brain abscess, and encephalitis were documented.

Renal complications were also notable, with 3 cases observed, comprising individual instances of acute kidney injury (AKI), mucor nephropathy, and hydronephrosis.

Thrombosis emerged as another significant complication during hospitalization. Alongside the 3 cases of CVT and 1 case of cavernous sinus thrombosis, there were 3 cases of myocardial infarction (MI), 1 case of pulmonary thromboendarterectomy (PTE), and 1 case of superior ophthalmic vein thrombosis.

Furthermore, as depicted in Figure 1, two patients were admitted to the hospital with a chief complaint of diabetic ketoacidosis (DKA), while two other patients developed DKA during hospital stay.

Ultimately, the total number of mortalities was 15 (26%), with two of these cases not undergoing surgical debridement (4%). Notably, one of these two patients also did not receive antifungal medication. Among the fatalities, seven patients died from COVID-19-induced acute respiratory distress syndrome (ARDS) (47%), three succumbed to septic shock (20%), and two died due to massive PTE (13%). Another patient died from a MI (7%). In addition, one patient died from a SAH and another due to a massive infarction involving the right middle cerebral artery (MCA) and posterior cerebral artery (PCA) (7%).

## 4. Discussion

This study consisted of 57 patients with CAM, of whom 67% were male, with a mean age of 56.03 years. Ophthalmic signs were present in 84% of the cases, with periorbital edema being the most common symptom observed in almost half of the patients, followed by decreased visual acuity and orbital pain. Nasal symptoms and neurologic symptoms such as facial numbness and facial palsy were less frequent. The majority of patients (74%) had DM, followed by hypertension (47%), and a smaller percentage (11%) had a history of corticosteroid use. Antifungal therapy was administered to most patients, with 93% receiving amphotericin B and 75% treated with posaconazole. Surgical intervention, primarily FESS, was performed in 75% of the cases. Despite aggressive treatment measures applied when required, the mortality rate was 26%, with complications such as CNS involvement and thrombotic events contributing significantly to this outcome.

This study's demographic findings, including a predominance of male patients and a mean age in the mid-50s, are consistent with the reports by Hoenigl et al., where the majority of CAM patients were middle-aged men [7, 13]. Similarly, the high prevalence of ophthalmic signs and symptoms, particularly periorbital edema and vision loss, is a common finding across multiple studies, including those by Hussain et al., who noted that ophthalmic complications, particularly periorbital edema, are the hallmark of ROCM and also highlighted that visual disturbances, including vision loss and ptosis, are prevalent in CAM patients, indicating the aggressive angioinvasive nature of the infection. [14]. However, nasal symptoms were less frequent in this study compared with others, such as those by Garg et al., which emphasized nasal symptoms as an early sign of ROCM, especially in patients with poorly controlled diabetes, further reinforcing the diagnostic significance of these symptoms [15]. This could reflect variations in the severity of mucormycosis presentation across different populations.

Comorbidities, specifically DM, were a major risk factor, consistent with findings from multiple other reports. The 74% prevalence of diabetes mirrors the high rates similarly observed in studies by Dilek et al. [16] and Singh et al. [13, 16]. The pathophysiological mechanism linking diabetes to mucormycosis lies in hyperglycemia-induced acidosis, which impairs immune response and promotes fungal proliferation. Furthermore, Singh et al. [13] found that uncontrolled diabetes significantly worsened outcomes in CAM patients, supporting the view that glycemic control is critical in preventing mucormycosis in the general population as well as in COVID-19 patients.

Steroid use, present in 11% of the patients, was comparable to rates observed in other studies, though the widespread use of corticosteroids in COVID-19 management is linked to an overall increase in mucormycosis cases [7, 14–16]. The widespread use of steroids during the pandemic may have inadvertently contributed to the rise in mucormycosis cases, as noted by Wiersinga et al. [17] who discussed how immunosuppressive therapies in COVID-19 could increase the risk of secondary infections like mucormycosis by increasing the blood glucose level and decreasing phagocytic activity of WBC. It is notable, however, that the actual rate of corticosteroid use may be higher than reported, as these data were based on self-reports from patients or their relatives rather than documented prescriptions due to the lack of a centralized drug usage database in Iran.

Furthermore, the relatively high prevalence of hypertension (47%) is compatible with reviews by Hussain et al. [14] and Dilek et al. [16], who identified hypertension as a frequent underlying condition in mucormycosis patients. While the exact link between hypertension and mucormycosis is less clear, it is plausible that hypertension, as a component of metabolic syndrome, contributes to overall immune dysfunction and worsens patient outcomes.

In this study, treatment for CAM primarily involved a combination of antifungal therapy and surgical intervention, which are critical for managing the infection's aggressive nature. Amphotericin B remained the first-line antifungal, administered to 93% of the patients, aligns with findings by Honavar and others, who also reported high rates of amphotericin B use in CAM patients [10]. The use of combination therapy with posaconazole was also prevalent (75%), in line with reports from Kulendra et al., who noted its efficacy in combination with other antifungal agents [18, 19].

However, surgical intervention played an equally vital role, with 75% of the patients undergoing sinus and nasal surgery, including FESS, which was the most frequent procedure (61%), consistent with the recommendations in other studies [13, 18]. Surgical debridement is essential for reducing the fungal burden and improving survival outcomes, especially in cases where mucormycosis is localized to the sinuses, orbit, or brain [20]. Other significant procedures included maxillectomy (16%), ethmoidectomy (11%), and middle turbinectomy (7%), which reflect the need for extensive debridement in patients with severe sinus and nasal involvement. In addition, 12% of the patients required orbital surgeries, including orbitotomy and vitrectomy, highlighting the aggressive angioinvasive nature of the infection and the need for more extensive surgical interventions in cases where the orbit or brain is affected [7].

Comparing these results with other studies, some similar trends can be seen. In the studies by Honavar [10] and Alekseyev et al. [19], surgical intervention was shown to significantly improve survival rates, especially when combined with early antifungal therapy. In those studies, FESS was also the most commonly performed procedure for ROCM, demonstrating its critical role in managing localized infection. In addition, Nazari et al. in their systematic review of CAM in Iran also emphasized the necessity of early and aggressive surgical debridement to improve patient outcomes [12].

However, it is worth noting that in some studies, like that of Hoenigl et al., surgical intervention was associated with higher survival rates, but the mortality remained high, particularly in patients with CNS involvement (38.32%) [7]. In current study, CNS involvement was present in 25% of the patients, contributing to a mortality rate of 26%, which is slightly lower than in these other reports but still underscores the danger of delayed or insufficient intervention. Furthermore, retrobulbar amphotericin B injections, performed in 28% of our cases, are another therapeutic strategy seen in other CAM studies, although their effectiveness is still a subject of ongoing research [21].

CNS involvement and thrombotic events were observed in 14 patients in the form of cerebral intravascular complications and in one case each of PTE and superior ophthalmic vein thrombosis. These findings highlight the angioinvasive nature of mucormycosis, which is exacerbated by the prothrombotic state induced by COVID-19 [22]. Also, the use of corticosteroids, while crucial in managing severe COVID-19, further increases the risk of such angioinvasive infections, as highlighted by Wiersinga et al. [17] This dual challenge of managing COVID-19 while preventing opportunistic fungal infections like mucormycosis underscores the importance of a balanced therapeutic approach.

An important point to emphasize is that 8 patients (14%) did not undergo any surgical intervention, either due to poor prognosis or contraindications for surgery. As expected, the mortality rate in this subgroup was significantly higher, with 2 patients dying without any surgical or antifungal treatment, further reinforcing the necessity of early and comprehensive surgical management.

It should be noted that this research may have some missing data, as it was conducted retrospectively, relying on information collected for the treatment of patients rather than evaluating them as part of a research case series. For example, pH levels, an important predisposing factor, and serum iron levels were unavailable for any patient, despite high iron levels being recognized as a predisposing factor for mucormycosis [1, 13].

In conclusion, this study highlights the importance of early diagnosis and aggressive treatment of mucormycosis, particularly in patients with metabolic disorders or immunosuppression, such as diabetes or those receiving corticosteroid therapy. Although COVID-19 is no longer a widespread concern, the lessons learned about managing mucormycosis remain highly relevant for future outbreaks. Prompt initiation of antifungal therapy and surgical debridement continue to be the cornerstone of treatment. Further research is essential to refine protocols and improve survival outcomes for patients with CAM in similar scenarios.

### 4.1. Limitations

This study was conducted retrospectively, and an evaluation of additional variables was not possible. All patients assessed were admitted and diagnosed with concurrent COVID-19 and mucormycosis infection during the pandemic. Unfortunately, data were not available regarding patients with mucormycosis infection in the pre-COVID-19 era, which prevented us from conducting a comparison of the predisposing and prognostic factors of these two populations.

## Figures and Tables

**Table 1:** Frequency of complains on arrival per patients.

**Ophthalmic disorders (*n* = 48 (84%))**	

Periorbital edema	26 (46%)
Decreased visual acuity	16 (28%)
Orbit pain	10 (18%)
Ptosis	7 (12%)
Proptosis	4 (7%)
Blindness	3 (5%)
Frozen eye	3 (5%)
Corneal ulcer	1 (2%)
Pussy discharge from eye	1 (2%)

**Nasal disorders *n* = 11 (19%)**	

Nasal discharge	5 (9%)
Epistaxis	5 (9%)
Paranasal pain	1 (2%)

**Neurologic disorders *n* = 10 (18%)**	

Facial numbness	6 (11%)
Facial palsy	2 (4%)
Seizure	1 (2%)
Confusion	1 (2%)
Slurred speech	1 (2%)

**Others**	

Headache	11 (19%)
Oral ulcer	5 (9%)
Fever	3 (5%)
Diabetic ketoacidosis	2 (4%)
Deep vein thrombosis	1 (2%)
Bloody sputum	1 (2%)
Abdominal pain	1 (2%)

**Table 2:** Clinical syndrome.

Rhino-orbital	42 (74%)
Rhino-orbital-cerebral	14 (25%)
Sinusitis alone	1 (2%)

**Table 3:** Frequency of pre-existing comorbidities per patients.

Diabetes	42 (74%)
Hypertension	27 (47%)
Ischemic heart disease	10 (18%)
Recent corticosteroid consumption due to COVID-19	6 (11%)
Hyperlipidemia	5 (9%)
Cancer	5 (9%)
Kidney disorder (single kidney–kidney transplant–other injury)	5 (9%)
Chronic obstructive pulmonary disease	2 (4%)
Cerebrovascular disease	2 (4%)
Rheumatoid arthritis	1 (2%)
Pulmonary hypertension	1 (2%)
Cirrhosis	1 (2%)
Chemical warfare injury	1 (2%)
Hypothyroidism	1 (2%)
No significant medical history	9 (14%)

**Table 4:** Antifungal medication.

Amphotericin	53 (93%)
Posaconazole	43 (75%)
Caspofungin	12 (21%)
Fluconazole	3 (5%)
Voriconazole	3 (5%)
Nystatin	2 (4%)
Clotrimazole	1 (2%)
No antifungal medication	2 (4%)

**Table 5:** Frequency of surgical interventions per patients.

Sinus and nasal surgery 43 (75%)	Functional endoscopic sinus surgery (FESS)	35 (61%)
	Maxillectomy	9 (16%)
	Ethmoidectomy	6 (11%)
	Middle turbinectomy	4 (7%)
	Nasal polypectomy	3 (5%)

Orbital surgery 7 (12%)	Orbitotomy	7 (12%)
	Vitrectomy	1 (2%)

Retrobulbar amphotericin injection	16 (28%)	

No intervention	8 (14%)	

**Table 6:** Complications during hospitalization.

**CNS extension**	

Cerebrovascular accident	4
Cerebral venous thrombosis	3
Subarachnoid hemorrhage	2
Aneurysm	2
Intraventricular hemorrhage	1
Carotid cavernous fistula	1
Cavernous sinus thrombosis (orbital apex stenosis)	1
Brain abscess	1
Encephalitis	1

**Kidney extension**	

Acute kidney injury	1
Mucor nephropathy	1
Hydronephrosis	1

**Other thrombosis**	

Myocardial infarction	3
Pulmonary thromboendarterectomy	1
Superior ophthalmic vein thrombosis	1

**Other complications**	

Diabetic ketoacidosis	2
Thrombocytopenia	2
Septic shock	2
Melena	1
Endotracheal tube actinobacteria	1
Heparin-induced thrombocytopenia	1
Lung collapse	1

## Data Availability

The data that support the findings of this study are available on request from the corresponding author. The data are not publicly available due to privacy or ethical restrictions.
